# Translation and Validation of the Chinese Version of Moral Courage Scale for Physicians

**DOI:** 10.5334/pme.1896

**Published:** 2025-11-14

**Authors:** Mingtao Huang, Qiuyu Huang, Yihang Shi, Hengxian Dong, Wenhong Dong, Guiru Xu

**Affiliations:** 1 School of Population Medicine and Public Health, Chinese Academy of Medical Sciences and Peking Union Medical College, Beijing, China; 2 Operating room, The Second Affiliated Hospital of Fujian Medical University, Quanzhou, China; 3 Department of colorectal surgery, National Cancer Center/ National Clinical Research Center for Cancer/Cancer Hospital, Chinese Academy of Medical Sciences and Peking Union Medical College, Beijing, China; 4 School of Foreign Languages, Renmin University of China, Beijing, China; 5 Department of Medical Quality Management, Fujian Medical University Union Hospital, Fuzhou, China; 6 School of Nursing, Fujian Medical University, Fuzhou, China

## Abstract

**Background:**

Moral courage is increasingly recognized as crucial for medical professionalism. This study aimed to translate and validate the Moral Courage Scale for Physicians into Chinese, evaluate its psychometric properties, and explore its applicability within the Chinese medical context.

**Methods:**

A cross-sectional study involving 425 licensed physicians across Mainland China was conducted between February and April 2025. Participants completed an online survey including demographic information and the Chinese version of MCSP. Data were analyzed using EFA, CFA, and Cronbach’s alpha.

**Results:**

The Chinese MCSP demonstrated strong psychometric properties, including high internal consistency (Cronbach’s alpha = 0.935) and a clear single-factor structure explaining 65.94% of the variance. CFA supported good model fit (χ^2^/df = 3.167, GFI = 0.958, RMSEA = 0.071, CFI = 0.978, TLI = 0.971). Physicians in China reported high levels of self-assessed moral courage, consistent with previous international studies.

**Conclusion:**

The validated Chinese MCSP is reliable and valid for assessing moral courage among Chinese physicians. It provides a robust tool for future research, medical education, and ethical training programs to enhance physician professionalism and patient care quality in China.

## 1 Introduction

For centuries, an essential component of the physicians’ professional oath has been the promise to place patients’ interests above all else [1]. Generations of doctors have bravely upheld this principle – even at personal risk, such as caring for contagious patients during epidemics [2]. However, contemporary clinical practice presents new ethical dilemmas that make honoring this oath increasingly challenging. Physicians may be forced to ration scarce resources among equally needy patients [3], face the risk of death or harm when providing treatment [4], decide whether to disclose a serious medical error to a patient [5], or speak out against unethical or unsafe practices by colleagues [6]. When clinicians feel unable to act according to their moral ideals in such situations, they often experience moral distress – characterized by negative emotions like guilt, shame, and self-blame [7].

Research suggests that moral courage can be a crucial antidote to moral distress [8]. Moral courage can be defined as the voluntary willingness to stand up for and act on one’s ethical beliefs despite barriers that may inhibit the ability to proceed toward right action [9]. In practice, moral courage manifests in two main ways: standing up to others when witnessing wrongdoing, and owning up to one’s own mistakes or ethical obligations [10].

Courage has long been examined in moral philosophy as a virtue. In Aristotelian virtue ethics, courage is one of the cardinal virtues – a mean between cowardice and recklessness – that must be cultivated through practice and habituation [11]. China’s cultural context differs markedly from that of Western countries. Concepts of virtue and duty in Chinese culture are deeply influenced by Confucian values. Confucian ethics similarly esteem courage (*yong*) but emphasize that it must be guided by righteousness (*yi*) to be morally admirable [12]. These philosophical frameworks highlight that moral courage is not an innate trait bestowed upon a few heroes, but a developed virtue: it can be strengthened through education, reflection, and experience [13]. Modern educators echo this view – encouraging training programs to instill virtues like courage, honesty, and integrity in professionals over time [14]. Empirical evidence supports that ethics education and positive role-modeling can indeed enhance moral courage in healthcare providers [15].

Within the medical profession, moral courage is increasingly recognized as a core component of professionalism and physician identity. It is considered a professional expectation that clinicians will uphold ethical standards even under pressure [16]. Importantly, moral courage appears to play a role in the formation of healthcare providers’ professional identity – the integration of moral values into one’s self-concept as a ‘good physician’. In Confucian ethics, maintaining harmony and deference to authority are highly emphasized [17], which can possibly affect how and when doctors voice dissent or uphold principles. A recent cross-sectional study showed that nurses with higher moral courage tend to report a stronger sense of professional identity. Conversely, lack of moral courage has been linked with moral disengagement and erosion of professional integrity [8]. Thus, fostering moral courage is pertinent not only for resolving immediate ethical dilemmas but also for shaping resilient, principled physicians over the long term.

Despite the clear importance of moral courage, assessing it reliably has been a challenge, largely due to a lack of physician-specific measurement tools. When this gap was first being addressed, existing instruments were often inadequate for evaluating this specific construct within the medical profession. For instance, some scales were designed for other healthcare professionals, such as the Nurses’ Moral Courage Scale, which addresses scenarios and contexts unique to the nursing profession [18]. Furthermore, while other scales of moral courage existed, they were typically developed for and validated in non-clinical settings, limiting their applicability to the unique ethical dilemmas faced by doctors [19]. This notable gap highlighted the need for a tailored instrument. To address this, in 2016, Martinez *et al*. developed the Moral Courage Scale for Physicians (MCSP), a nine-item survey to quantify medical trainees’ propensity to act courageously in clinical ethical situations [9]. We chose the MCSP for this study because it is uniquely grounded in medical scenarios and had demonstrated strong reliability among U.S. physician trainees. Since then, the MCSP has been applied in a few other contexts [20]. However, no Chinese-language version of the MCSP was available, hindering research and educational efforts around moral courage in China’s medical community.

China’s healthcare system and cultural context differ in many ways from those of Western countries where the MCSP was developed. It is therefore important to examine whether the construct of ‘moral courage’ as captured by the MCSP is equally relevant and valid among Chinese physicians. Developing a Chinese MCSP could fill a critical gap by providing educators and researchers with a tool to assess moral courage in Chinese medical settings. Such a tool could enable baseline measurements of physicians’ moral courage, comparisons with international data, and evaluations of interventions aimed at strengthening clinicians’ courage in the face of ethical adversity. Ultimately, understanding and bolstering moral courage among Chinese physicians could help improve ethical decision-making, reduce moral distress, and enhance patient care quality in China.

**Study Purpose:** In this context, the present study set out to translate and validate the MCSP for use in China. Specifically, we aimed to (1) produce a culturally adapted Chinese version of the Moral Courage Scale for Physicians, and (2) rigorously evaluate its psychometric properties in a sample of Chinese physicians. We also explored how Chinese physicians score on moral courage and discussed the implications for medical ethics education and clinical practice. Our goal is to contribute to the global discourse on moral courage by adding evidence from China, and to provide a practical instrument that can guide future training and policy efforts to foster moral courage in healthcare professionals.

## 2 Methods

### 2.1 Participants

This cross-sectional study was conducted from February to April 2025 to evaluate the Chinese version of the MCSP. A convenience sampling method was used to recruit licensed physicians across Mainland China as study participants. Inclusion criteria for participation were: (1) holding a valid practicing physician qualification certificate, and (2) voluntary participation. To ensure the reliability of the factor analysis results, a minimum sample size of 50 participants was required for exploratory factor analysis (EFA) [21], and at least 200 participants for confirmatory factor analysis (CFA) [22]. A total of 440 questionnaires were collected, and 425 were valid, resulting in an effective recovery rate of 96.59%.

### 2.2 Measures

#### General information

The general demographic information was collected using a researcher-developed questionnaire, which was created after a review of the literature and through group discussions. This questionnaire includes 18 items, including gender, education level, professional title, and other relevant characteristics.

#### MCSP

MCSP [9] is a reliable tool used to assess a physician’s self-reported moral courage. The scale consists of 9 items rated on a 7-point Likert scale, ranging from 1 (strongly disagree) to 7 (strongly agree). Total scores are transformed to a scale of 0 (indicating the lowest moral courage) to 100 (indicating the highest moral courage) using the formula: (average score – 1) × (100/6).

### 2.3 Scale translation and cross-cultural adaptation procedure

After obtaining permission from the original authors, the MCSP was translated into Chinese using the classical «backward and forward» translation procedure, following a modified Brislin translation model [23]. Two independent bilingual Chinese native translators performed the forward translation of the items from English to Chinese. After discussion and revision by all authors and translators, a consensus forward version of the Chinese scale was developed.

Subsequently, the reconciled forward Chinese version was back-translated into English by two independent bilingual translators who were blind to the original English version of the scale. In the third step, the original and back-translated English versions were compared to ensure semantic equivalence. The comparison process was conducted in accordance with the cross-cultural adaptation guidelines [24]. The versions were then reviewed by a panel of three experts to ensure linguistic accuracy and cultural relevance. This expert panel included two senior physicians (one in internal medicine and one in surgery) and a professor of medical ethics, each with over 10 years of experience in their field. Their diverse professional backgrounds ensured that the translated items retained their clinical clarity, educational relevance, and ethical appropriateness.

Finally, cognitive interviews were conducted with 10 Chinese physicians (5 male, 5 female; representing internal medicine, surgery, pediatrics, and primary care) to evaluate the translated scale’s clarity and cultural relevance, particular attention was paid to resolving potential ambiguities in the original wording. Each participant completed the draft Chinese MCSP while voicing their thoughts, followed by a debriefing interview. Participants were asked what each item meant to them and whether any wording was confusing or not applicable in the Chinese context. Feedback from these interviews indicated that the items were generally well-understood. Minor wording adjustments were made based on suggestions. For example, A1 ‘I do what is right for my patients’ was refined to ‘I do what I think is right for my patients’. In addition, we use the example of ‘opposition from superiors’ to explain the phrase ‘opposing social pressures’. The input from the 10 physicians helped ensure the translated scale was culturally appropriate and easily comprehensible for Chinese physicians.

### 2.4 Data collection procedure

Data for this study were collected through an online survey platform, Wenjuanxing (survey link: https://www.wjx.cn/vm/PGXe49z.aspx#).

Before beginning the survey, participants were provided with an introductory section that outlined the definition of moral courage, the purpose and significance of the study, and instructions on how to complete the questionnaire. The first page of the survey contained an informed consent statement, clearly explaining the voluntary nature of participation, the confidentiality of responses, and the right to withdraw at any time without consequences. Participants who agreed to take part in the study had to click the ‘Agree’ button to proceed to the questionnaire, while those who did not consent could select ‘Disagree’ and exit the survey without providing any responses.

The questionnaire was structured into multiple sections, including demographic information and the MCSP items. The estimated completion time for the survey was approximately 3 minutes. To ensure the quality of responses, mandatory response settings were applied to all key items, and duplicate submissions from the same IP address were restricted.

### 2.5 Statistical analyses

EFA and CFA were conducted to explore and verify the factor structure of the translated Chinese version of the MCSP. For EFA, the scale was considered suitable if the Kaiser-Meyer-Olkin (KMO) measure was greater than 0.6 and Bartlett’s test of sphericity was statistically significant (P < 0.05). CFA was performed using Amos 24.0 with maximum likelihood estimation (ML), which is appropriate for continuous and approximately normally distributed data. Model fit was evaluated using the χ^2^/df ratio, root mean square error of approximation (RMSEA), comparative fit index (CFI), Tucker–Lewis index (TLI), and goodness-of-fit index (GFI). A model fit was considered acceptable when χ^2^/df was < 5.0, RMSEA was < 0.08, and both CFI and TLI were > 0.90 [25]. All questionnaires included in the analysis were complete; cases with missing responses were excluded at the data screening stage, so no imputation procedures were necessary.

To assess the internal consistency reliability of the scale, Cronbach’s α coefficient was calculated, with values exceeding 0.7 considered acceptable for reliability [26]. A two-sided chi-square test was used to evaluate differences in demographic characteristics between the groups in the EFA and CFA stages. Independent t-tests were used to compare the total MCS scores and subscale scores across different physician demographics. All data were analyzed using SPSS 25.0 and Amos 24.0, with a significance level set at α = 0.05 (two-tailed).

## 3 Results

### 3.1 Demographic information

A total of 425 physicians participated. We randomly divided the sample into two independent datasets: 210 participants for EFA and 215 for CFA. Randomization was done by assigning computer-generated random numbers to each response and splitting at the median. Demographic characteristics are detailed in Table 1. The majority of participants were male (70.12%), aged 30–39 years (56.47%), and held a master’s degree (70.59%). Most participants had obtained their practicing physician qualification certificates between 1 and 10 years before to participating in the study (63.53%), were physicians-in-charge (46.12%), and worked in tertiary hospitals (73.18%). A majority (91.76%) were employed at comprehensive hospitals.

**Table 1:** Participants’ demographic characteristics (n = 425).


VARIABLE	CATEGORY	n	%

Gender	Male	298	70.12

	Female	127	29.88

Age	20–29	87	20.47

	30–39	240	56.47

	40–49	79	18.59

	50–59	19	4.47

Education	Associate degree	3	0.71

	Bachelor	61	14.35

	Master	300	70.59

	Doctorate	61	14.35

Years licensed	1–5	132	31.06

	6–10	138	32.47

	11–15	99	23.29

	≥16	56	13.18

Title	Resident physician	141	33.18

	Physician-in-charge	196	46.12

	Associate chief physician	66	15.53

	Chief physician	22	5.18

Hospital level	Tertiary	311	73.18

	Secondary	101	23.76

	First level	13	3.06

	Comprehensive	390	91.76

	Specialized (excl. psychiatric)	34	8

	Psychiatric	1	0.24

Department	Internal medicine	97	22.82

	Surgery	163	38.35

	Pediatrics	34	8

	Gynaecology & obstetrics	40	9.41

	Emergency	17	4

	ICU	6	1.41

	Anesthesiology	15	3.53

	Stomatology	8	1.88

	Other	38	8.94

Economic	Extremely difficult	5	1.18

	Some difficulties	37	8.71

	Basic needs met	188	44.24

	Relatively affluent	185	43.53

	Very affluent	10	2.35

Ethics course^1^	Yes	417	98.12

	No	8	1.88

Hospital ethics ed.^2^	Yes	388	91.29

	No	37	8.71

Self-learning ethics^3^	Yes	363	85.41

	No	62	14.59

Ethical dilemmas^4^	Always	23	5.41

	Often	109	25.65

	Sometimes	202	47.53

	Occasionally	91	21.41

	Never	10	2.35

Confidence^5^	Unconfident	38	8.94

	Neutral	146	34.35

	Confident	203	47.76

	Very confident	38	8.94

Stress from dilemmas^6^	Yes	246	57.88

	No	179	42.12

Seek help^7^	Always	13	3.06

	Often	84	19.76

	Sometimes	212	49.88

	Occasionally	105	24.71

	Never	11	2.59

Uphold morals^8^	Yes	411	96.71

	No	14	3.29


*Note:* ^1^Have you ever received medical ethics courses or related education? ^2^Has your hospital organized any educational activities related to medical ethics? ^3^Would you take the initiative to gain medical ethics knowledge? ^4^How often do you face ethical dilemmas in your work? ^5^How do you evaluate your ability to handle ethical dilemmas? ^6^Have you ever felt anxious or stressed due to moral dilemmas in your work? ^7^When faced with moral dilemmas, do you make decisions by seeking help from colleagues or superiors? ^8^Do you believe that you will always uphold your moral beliefs throughout your career?

### 3.2 EFA

**Figure 1:**
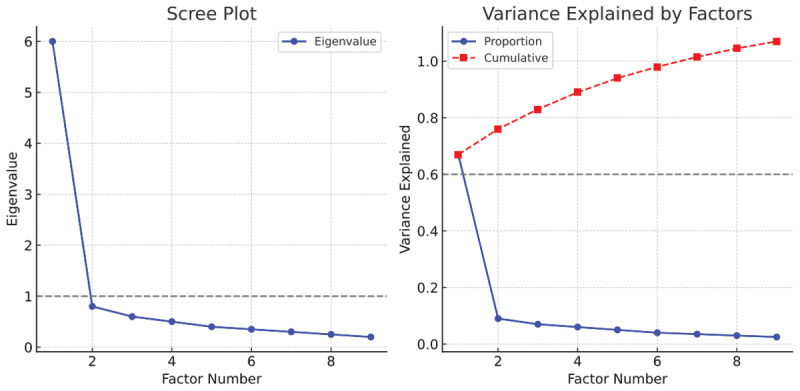
Scree plot from the exploratory factor analysis showing one-factor solution.

The KMO measure was 0.946, and Bartlett’s test of sphericity was significant (χ^2^ = 2708.120, P < 0.001), indicating that the data were suitable for factor analysis. One factor was extracted with an eigenvalue of 5.934, explaining 65.935% of the variance (Table 2). Factor loadings for items ranged from 0.733 to 0.906, indicating strong loadings and acceptable communalities (Table 2). The scree plot further confirmed the one-factor solution (Figure 1).

**Table 2:** Exploratory factor analysis results for the Chinese MCSP.


ITEM	EIGENVALUE	VARIANCE (%)	CUMULATIVE VARIANCE (%)	FACTOR LOADING	COMMUNALITY

A1	5.934	65.935	65.935	0.733	0.538

A2	0.580	6.441	72.376	0.906	0.822

A3	0.525	5.834	78.210	0.785	0.616

A4	0.485	5.391	83.602	0.841	0.708

A5	0.393	4.367	87.969	0.802	0.643

A6	0.329	3.658	91.627	0.734	0.539

A7	0.309	3.435	95.062	0.847	0.718

A8	0.286	3.179	98.242	0.783	0.613

A9	0.158	1.758	100.000	0.859	0.738


*Note:* Item codes A1–A9 correspond to the 9 scale items; all items loaded on a single factor.

### 3.3 CFA

**Figure 2:**
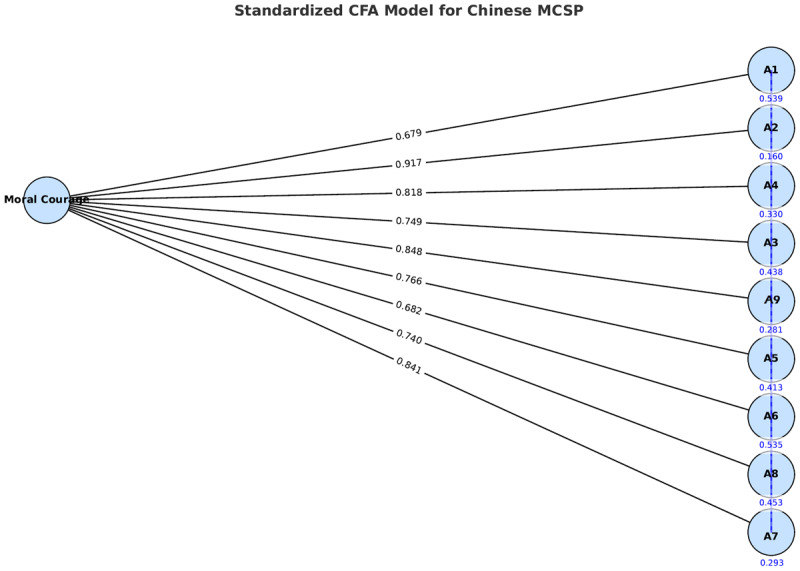
Standardized confirmatory factor analysis (CFA) model of the Chinese Moral Courage Scale for Physicians (MCSP).

CFA results indicated good model fit: χ^2^/df = 3.167, GFI = 0.958, AGFI = 0.930, RMR = 0.043, RMSEA = 0.071, CFI = 0.978, NFI = 0.969, and TLI = 0.971 (Table 3). The CFA model diagram illustrates the relationships among the items clearly (Figure 2).

**Table 3:** Confirmatory Factor Analysis Model Fit Indices.


FIT INDEX	VALUE	RECOMMENDED CRITERIA

χ^2^/df	3.167	<3.0

GFI	0.958	>0.90

AGFI	0.930	>0.90

RMR	0.043	<0.05

RMSEA	0.071	<0.08

CFI	0.978	>0.90

NFI	0.969	>0.90

TLI	0.971	>0.90


The latent variable ‘Moral Courage’ is displayed on the left. Singleheaded arrows denote standardized factor loadings to the nine observed indicators (A1–A9; black labels). Each indicator has a residual (unique) variance term (blue values inside small circles) with a oneheaded arrow pointing to the indicator. No residual covariances were freely estimated in the final model. All coefficients are standardized.

### 3.4 Reliability Analysis

The internal consistency reliability of the Chinese version of the MCSP was excellent, with an overall Cronbach’s alpha of 0.935. Item-total correlations ranged from 0.668 to 0.869, and the deletion of any item did not significantly increase Cronbach’s alpha, indicating high reliability and internal consistency of the scale (Table 4).

**Table 4:** Reliability Analysis (Cronbach’s Alpha).


ITEM	CORRECTED ITEM-TOTAL CORRELATION	CRONBACH’S ALPHA IF ITEM DELETED

A1	0.668	0.932

A2	0.869	0.919

A3	0.725	0.928

A4	0.790	0.924

A5	0.743	0.927

A6	0.669	0.931

A7	0.795	0.924

A8	0.722	0.928

A9	0.811	0.923


Overall, the results supported the validity and reliability of the Chinese version of the MCSP for evaluating moral courage among physicians in China.

## 4 Discussion

### 4.1 Chinese MCSP is highly reliable

In this study, we successfully translated and validated the MCSP in a Chinese context. The key findings are that the Chinese MCSP retains a unidimensional factor structure, demonstrates excellent reliability, and Chinese physicians tend to score very high in self-reported moral courage. These results closely mirror the characteristics of the original MCSP and its use in other countries, reinforcing the idea that the core construct of moral courage is a universally relevant trait in the medical profession.

The fact that a single factor emerged, explaining about 66% of the variance, and that CFA confirmed a good fit for a one-factor model, indicates that Chinese physicians conceptualize the various elements of moral courage as part of one overarching quality. This unidimensionality is consistent with Martinez et al.’s [9] initial development work and with a recent Turkish adaptation that also found one factor with α = 0.91 [20]. Our reliability (α = 0.935) was even slightly higher, suggesting the Chinese-translated items cohered very strongly. From a psychometric standpoint, this provides robust evidence for construct equivalence: the translated scale is measuring the same construct as intended. We did not find any need to drop items for the Chinese version; all original items loaded well. This outcome is encouraging because it implies that the essence of moral courage transcends language and culture – at least among physicians.

In terms of overall scoring tendencies, Chinese physicians in our sample reported a relatively high degree of self-assessed moral courage. Such uniformly high item scores are similar to those seen in the original MCSP validation [9], indicating a ceiling tendency where physicians generally perceive themselves as behaving morally courageously. In summary, our key findings are that the Chinese MCSP is highly reliable, has a unidimensional factor structure, and yields high self-reported scores on moral courage among Chinese physicians, laying a solid foundation for further use of this scale in China.

### 4.2 Implications for Theory and Practice

The findings from this study have significant implications for both the theoretical understanding of moral courage and its practical application in medicine. Theoretically, our successful validation of the MCSP in the Chinese language and context supports the notion that moral courage is a universally relevant construct in the medical profession. The consistency in the scale’s factor structure across cultures suggests that the core elements of moral courage – steadfast ethical agency, perseverance in the face of threats, commitment to moral values, and altruistic intent [27] – cohere into a single attribute that physicians recognize, regardless of cultural background. This adds evidence to the existing theory that moral courage is a distinct and measurable component of a healthcare professional’s character [9]. Moreover, our results bolster the argument from virtue ethics that moral virtues are not merely innate traits but can be identified and potentially cultivated in individuals. As Aristotle suggested and modern educators echo, such virtues can be taught and strengthened through practice and education [9]. In addition, the observed trends align with developmental theories, suggesting professional moral formation evolves with mentorship and exposure to ethical challenges. This implies that our understanding of moral courage must account not only for personal traits but also for the developmental and contextual factors that shape ethical behavior over a physician’s career.

Practically, these findings highlight the critical importance of fostering moral courage in healthcare settings and offer a validated tool to do so in China. The Chinese version of the MCSP can serve as a diagnostic instrument to identify strengths and gaps in moral courage within physician groups. For example, if certain subgroups (such as less experienced doctors) have slightly lower scores, targeted support or training can be provided to bolster their confidence in acting ethically. Our results, together with prior research, suggest that assessing and cultivating moral courage should be an educational and institutional priority [27]. The convergence of Chinese physicians’ responses with international patterns also means that interventions used successfully elsewhere are theoretically likely to be effective in China as well.

In practical terms, upholding high moral courage in the workforce can mitigate ethical complacency and reduce moral conflicts. Physicians who score high on moral courage are more likely to take appropriate action even when it is difficult. This bodes well for addressing issues like medical errors, informed consent, and patient advocacy in Chinese hospitals. Conversely, recognizing the contextual barriers highlighted above, healthcare leaders must also work to reduce the external risks that currently make moral courage costly to exercise. In doing so, they create an environment where the admirable levels of moral resolve reported by physicians in our study can more freely translate into ethical actions. Thus, the implications of our findings are twofold: they advance the scholarly understanding that moral courage is a reliable, cross-culturally valid construct, and they call for concrete efforts in practice to nurture and harness this quality for better healthcare outcomes.

### 4.3 Applications of the Chinese MCSP

The validated Chinese MCSP opens several avenues for application in medical education, clinical practice, and research.

The Chinese MCSP can be integrated into medical school and residency curricula as both an educational and evaluative tool. Educators might administer the MCSP to medical students or trainees to self-assess their moral courage at different stages of training. This can help identify learners who may need additional support in developing ethical confidence. Incorporating scenario-based learning followed by MCSP assessments could allow students to reflect on their willingness to act morally under pressure. This is significant because moral courage can be nurtured through teaching and practice [18]. Ultimately, integrating the MCSP in medical education promotes reflection and growth in an attribute that is as important as clinical knowledge for the making of a good physician.

For practicing physicians and healthcare teams in China, the MCSP offers a practical means to guide and enhance ethics training programs. Hospitals could use the MCSP as part of regular staff development or quality improvement initiatives to gauge the ethical climate and courage levels in different departments. They can also apply this scale to benchmark and cultivate moral courage as a key component of clinical excellence. However, many contextual factors can influence score shifts over time. Thus, any longitudinal use of the MCSP should be interpreted with caution, and supplemented by qualitative insights where possible.

The Chinese MCSP enables new research into how moral courage interacts with other critical aspects of healthcare practice. Researchers can now quantitatively examine whether physicians with higher moral courage experience lower levels of moral distress or burnout. In summary, the Chinese MCSP is a powerful tool for advancing research on the interplay between individual virtues and systemic factors in healthcare. By applying it in studies of moral distress, resilience, and decision-making, we can better understand and ultimately improve the moral fabric of medical practice in China.

### 4.4 Cultural considerations in the interpretation and expression of moral courage

Cultural context plays a pivotal role in shaping how individuals interpret the items of the MCSP and express moral courage in clinical practice. Although the Chinese MCSP demonstrated strong psychometric equivalence to the original scale, it is important to acknowledge that cultural factors may shape both the understanding of moral courage and its behavioral manifestations. In a Confucian-influenced society, there is a strong emphasis on respecting authority and maintaining social harmony [17]. This linkage may influence how Chinese physicians interpret items such as item 1, where ‘opposing pressures’ could be understood more as challenges to hierarchical authority. Similarly, item 8 may be interpreted through the lens of collective responsibility. In a collectivist culture, ‘risk’ is not only personal but may also encompass the potential consequences for one’s team or institution. This broader sense of responsibility could make physicians more cautious in exercising overt moral dissent, favoring strategies that protect both patient interests and institutional stability. As a result, while the scale items were retained verbatim in translation, their behavioral correlates may manifest differently in practice. Recognizing these influences enhances the cultural relevance of the scale and supports more nuanced cross-cultural comparisons of moral courage in the medical profession.

## 5 Limitations

Despite its strengths, this study has several limitations. First, we did not assess test–retest reliability, so the stability of the MCSP over time remains unconfirmed. Future studies should evaluate the Chinese MCSP’s score consistency over repeated administrations. Second, all data were self-reported, introducing potential response biases. Third, the sample, while geographically diverse, was not randomly selected, which may affect generalizability of the findings.

## 6 Conclusions

This study successfully validated the Chinese version of the MCSP, confirming its strong reliability and validity. The findings underscore the universality of moral courage in medical practice while highlighting unique aspects of its expression within Chinese cultural and systemic contexts. The Chinese MCSP offers a valuable instrument for assessing moral courage, facilitating targeted educational interventions, and supporting ethical decision-making in clinical settings. Its widespread adoption in medical training and hospital management could foster an environment that consistently empowers physicians to act ethically, ultimately benefiting patient care and professional integrity.

## 7 Learning Points

The Chinese version of the MCSP has strong reliability and validity, making it suitable for research and practical applications in China.

Moral courage among physicians manifests similarly across cultures but is influenced by local sociocultural and systemic factors.

Routine assessment of moral courage using the MCSP can inform targeted ethics education and interventions, enhancing physicians’ ability to address ethical challenges.

## Data Accessibility Statement

Data generated in this study are available from the corresponding author upon request.

## Appendix

### Original version

1. I do what is right for my patients, even if I experience opposing social pressures (e.g., opposition from senior members of the healthcare team, medical guidelines, etc.).
2. I use a guiding set of principles from my profession to help determine the right thing to do for my patients.
3. My patients and colleagues can rely on me to exemplify moral behavior.
4. I do what is right for my patients because it is the ethical thing to do.
5. I go above and beyond what is required to do what is right for my patients.
6. When faced with ethical dilemmas in patient care, I consider how both my professional values and my personal values apply to the situation before making decisions.
7. When I do the right thing for my patients, my motives are pure.
8. I do what is right for my patients, even if it puts me at risk (e.g., legal risk, risk to reputation, etc.).
9. I am determined to do the right thing for my patients.

### Chinese version

1. 即使面临社会压力（如上级的反对），我依然会为我的患者做我认为是正确的事。
2. 我会使用职业道德规范和医学原则进行帮助和判断，为我的患者做我认为是正确的事。
3. 我的患者和同事可以信赖我，因为我以身作则并且践行道德行为。
4. 为我的患者做我认为是正确的事，因为这是合乎伦理的。
5. 我会超越职责要求，为患者做我认为是正确的事。
6. 当我面对患者治疗中的伦理困境时，我会综合考虑自己的职业价值观与个人价值观，才会做出决策。
7. 当我为患者做我认为是正确的事情时，我的出发点是纯粹的、无私的。
8. 即使做我认为是正确的事可能影响我的个人声誉或让我面临法律纠纷，我仍会去做。
9. 我有坚定的决心，为我的患者做我认为是正确的事情。
